# Can Individualized Learning Plans in an advanced clinical experience course for fourth year medical students foster Self-Directed Learning?

**DOI:** 10.1186/s12909-016-0744-8

**Published:** 2016-09-01

**Authors:** Maribeth B. Chitkara, Daniel Satnick, Wei-Hsin Lu, Howard Fleit, Roderick A. Go, Latha Chandran

**Affiliations:** 1 Department of Pediatrics, Stony Brook Children’s, HSC T11-080, Stony Brook, NY 11794-8111 USA; 2 Department of Emergency Medicine, Mount Sinai West, NY USA; 3 Department of Preventive Medicine, Stony Brook Children’s, HSC T11-080, Stony Brook, NY 11794-8111 USA; 4 Department of Pathology, Stony Brook Children’s, HSC T11-080, Stony Brook, NY 11794-8111 USA; 5 Department of Medicine, Stony Brook Children’s, HSC T11-080, Stony Brook, NY 11794-8111 USA

**Keywords:** Individualized learning plans, Self-directed learning, Fourth year medical students

## Abstract

**Background:**

Residency programs have utilized Individualized Learning Plans (ILPs) to customize resident education while undergraduate medical education has not done so in a meaningful way. We discuss the use of ILPs within a fourth year medical school course to facilitate self-directed learning (SDL).

**Methods:**

At Stony Brook University School of Medicine, an ILP component was added to the Advanced Clinical Experience (ACE) course for fourth year students. Each completed an ILP outlining personal learning goals and strategies to achieve them. An adaptation of the Motivated Strategies for Learning Questionnaire (MSLQ) (Duncan T and McKeachie W, Educ Psych 40(2):117-128, 2005 and Cook DA et al., Med Ed 45:1230-1240, 2011) was used to measure success of ILPs in improving SDL. Qualitative data analysis was conducted on the ILPs and self-reflections.

**Results:**

Forty-eight students participated. Two of the four SDL sub-domains identified on the MSLQ showed improvement; self-efficacy (*p* = .001) and self-regulation (*p* = .002). ‘Medical Knowledge’ was the competency most frequently identified as an area of concentration (90 %) and professionalism was selected least frequently (4 %). A higher percentage (83 %) of students who reported complete achievement of their ILP goals also reported feeling better prepared for entering residency.

**Conclusions:**

ILPs improve SDL strategies among medical students and may serve as useful tools to help shape future learning goals as they transition to residency training.

## Background

Adaptive expertise and self-regulated learning skills of a physician are significant factors in knowledge acquisition and application in the patient care setting [1, 2]. The interplay between motivation and learning strategies is critical as the need to learn throughout one’s medical career can vary based on course requirements, career expectations, and clinical duties. Self-directed learning (SDL) is a process by which learners set personal learning goals, assess understanding, and close knowledge/skills gaps by acquiring or updating competencies as needed [3]. For medical students, the motivation to learn may be different from course to course; an elective versus a required course, and the learning strategies used may vary depending on the nature of the academic task; board exam versus observed structured clinical encounter. When planning an educational experience faculty should consider the diversity of learner interests, motivations, and learning strategies [4]. In 2010, the Carnegie Foundation for Advancement of Teaching called for reform in medical education [2]. Recognizing the important roles that intrinsic motivation and flexibility play in shaping the learning experience, they encouraged medical institutions to support curricula that standardize learning outcomes while individualizing the learning process.

An increasing number of residency programs have found success utilizing Individualized Learning Plans (ILPs) to customize education experiences with respect to interests, motivations, and learning strategies [2]. ILPs provide a specific format for trainees to identify content-based learning objectives, outline strategies for implementation of goals, and routinely re-assess progress. The essential components of an ILP include 1) reflection on long-term career goals and self-assessment of areas of strength and weakness, 2) goal generation, 3) development of plans/strategies to achieve the outlined goals, 4) progress assessment, and 5) revision of goals/plans based on self-assessment [2]. The ILP document should be fluid, with the learner continually revisiting goals and revising plans for progress. Goals can be short-term or long-term, knowledge-based; “I will focus on learning developmental milestones during my pediatric primary care week by performing developmental assessments on children of different ages”, or process-based; “I will practice my oral presentations on daily rounds and seek feedback at the end of each week from supervising physicians”.

Presently, the Accreditation Council for Graduate Medical Education (ACGME) requires that residents in Pediatrics construct ILPs annually during their training [5]. Accreditation standards from the Liaison Committee on Medical Education (LCME) ask medical schools to provide opportunities for students to identify individual learning needs and develop plans of action throughout the course of their education [6]. A national cross-sectional survey of pediatric and medicine-pediatric residents and residency program directors found success in reaching SDL goals was associated with a greater propensity to engage in lifelong learning behaviors [2]. These findings underscore the importance of developing SDL goals during undergraduate medical education; however, there is limited data on the use of the ILPs among medical students. In 2012, Shepard et al. published their experience with the implementation of a formal ILP program for sub-interns in Pediatrics and Internal Medicine [7]. Students in this study found the setting of personal learning objectives and strategies to be highly useful, and that the ILP helped them to accomplish more during their sub-internship rotation.

In 2012, Stony Brook University School of Medicine restructured the fourth year medical school curriculum in response to results from focus groups of graduating students as well as the results of the Association of American Medical Colleges (AAMC) Graduation Questionnaire, with a renewed emphasis on residency preparedness [8]. In this study, we describe the implementation of a formal ILP program for goal-setting in a novel clinical course for fourth year students. We anticipated that creating and completing ILPs as part of this clinical course would promote a significant increase in SDL behaviors among senior medical students.

## Methods

### Course description

As part of the restructured fourth year curriculum, a newly required course entitled Advanced Clinical Experience (ACE) was created at Stony Brook University School of Medicine. The ACE course is a four-week clinical course offered to students in fields related to their anticipated residency during the second half of their fourth year. At that point in the academic calendar, most fourth year students had completed the residency interview process and were beginning their preparation for transition to residency. The intention of the ACE course was to return students to the clinical arena of the field of their anticipated residency training. The structure of the clinical aspect of the course was similar to a typical sub-internship and dependent on the hospital setting and the learning goals of the students. For example, students in Pediatrics could choose to spend their clinical time on the pediatric inpatient unit, intensive care unit, neonatal intensive care unit, hematology-oncology unit or primary care setting. Students in the Internal Medicine ACE course had the choice of general medicine unit, intensive care unit, cardiac care unit, cardiology unit, oncology unit or primary care office.

During the ACE course, students were given the time equivalent to one week to independently pursue learning goals that could not be met in the schedule of their clinical work. Some independent learning experiences were spread across the entire four weeks of the course, while others were concentrated to a single week. For example, a trainee entering the field of Pediatrics who wished to strengthen their diagnostic skills regarding rashes might spend time with a pediatric dermatologist. Another student might use that time to round with a radiologist or to review electrocardiograms with a cardiologist.

In order to maximize success in the achievement of their learning goals, students were provided with a self-study module that included literature regarding the creation of ILPs and an ILP template. ACE students were instructed to consider the six ACGME core competencies and use the SMART (specific, measurable, achievable, realistic, timely) criteria when creating their learning goals [7, 9]. ACE site directors met with students prior to the initiation of the course to ensure that outlined objectives were appropriately specific to the learner, measurable, attainable and realistic in terms of task complexity. Limitations of time and available resources during the course were also considered as part of goal-setting. Students met with faculty mentors periodically throughout the ACE course to discuss progress and achievements in regard to their ILP goals. At the completion of the four weeks, students were asked to write a brief reflective essay regarding their experience in the ACE course and the process of setting of personal learning goals.

### Participants

During the 2012–2013 and 2013–2014 academic years, 256 fourth year medical students participated in the ACE course across all specialties. The convenience sample for our study was drawn from students who completed the Pediatrics or Internal Medicine ACE during this time period. Students were informed that participation is voluntary and that participation in the study or lack thereof would not affect his/her course grade or academic standing in the school.

The Stony Brook University School Institutional Review Board reviewed and approved this study as exempt (IRB # 410136–4). Written informed consent was obtained from all participants.

### Data sources and analysis

Data for this study were collected from four sources: the Motivated Strategies for Learning Questionnaire (MSLQ) [4, 10] student self-reported surveys, the ILPs, and student end-of-course reflective essays. The MSLQ is a validated measurement tool used to assess student’s self-directed learning beliefs and strategies, and was adapted for use among medical students. The adapted MSLQ contained 31 questions with a 7-point Likert scale (1 = not at all true of me, 7 = very true of me) and was administered before and after the ACE course. The MSLQ assessed motivational beliefs (self-efficacy and intrinsic value) and learning strategies (cognitive strategy use and self-regulation). Motivational beliefs include self-efficacy, an individual’s belief in his/her ability for success, and intrinsic value, the degree to which one studies material for the purpose of mastery [10]. Learning strategies include cognitive strategy use, the degree to which a student uses basic and complex strategies for processing information (e.g. rehearsal, elaboration, organization) and self-regulation, the ability to control effort when faced with distractions or boring tasks [4]. A paired-samples *t*-test was used to compare mean changes from before to after the ACE course on student responses to the MSLQ for each sub-domain of self-directed learning.

Additionally, students were given a survey that asked about their ILPs and preparedness for residency. The survey was developed by one of the authors (MBC) and was comprised of 4 questions using a 5-point scale. Specifically, the survey asked how prepared they felt to begin their residency program before and after completing the ACE course. At the end of the course, students were also surveyed on: (a) how helpful writing the ILP was in structuring his/her learning goals for the course (1 = not helpful at all, 5 = extremely helpful) (b) how helpful the ILP was in organizing his/her thoughts and goals regarding his/her career (1 = not helpful at all, 5 = extremely helpful) and (c) whether they were able to achieve his/her ILP goals and objectives (1 = not at all, 2 = partially; 3 completely). These results were analyzed using chi-squared tests.

Upon completion of the course, students wrote a self-reflection based on prompts pertaining to their clinical experience and ILP goals. Students were asked to identify: (a) what he/she would have done differently with his/her ILP; (b) whether or not he/she was able to meet his/her ILP goals; (c) the challenges he/she encountered in achieving his/her ILP goals; (d) how he/she overcame these challenges; and (e) what they have learned from this experience that will help him/her with his/her next step. Two of the authors (DS and W-HL) independently reviewed the ILPs and coded the students’ reflections and agreed on a single set of codes. A trustworthiness check by the other authors (MBC, RG, HF, and LC) using the coding schema on selected texts followed the initial coding by the two coders (DS and W-HL). A final analysis was then conducted and re-confirmed by all authors.

## Results

Of the 84 students enrolled in the selected ACE courses during the 2012–2013 and the 2013–2014 academic years, 48 students volunteered to participate. (*n* = 48; 57.14 % participation rate); twenty-three were in the Internal Medicine ACE (48 % of the 48 participating students) and twenty-five were in the Pediatrics ACE (52 % of the 48 participating students) (see Table 1 for details). There was significant improvement in two of the four SDL sub-domains identified on the MSLQ; self-efficacy (*p* = .001), self-regulation (*p* = .002), intrinsic value (*p* = .42), and cognitive strategy use (*p* = .10) (see Table 2 for details).

**Table 1:** Characteristics of participating students enrolled in the pediatric and medicine ACE course, AY2012-13 & AY2013-14

ACE course	Overall	Pediatrics	Medicine
Participating Students, no. (%)	48 (100 %)	25 (52 %)	23 (48 %)
Female Gender, no. (%)	23 (48 %)	15 (60 %)	8 (35 %)

**Table 2:** The Motivated Strategies for Learning Questionnaire (MSLQ) results of students pre and post the ACE course, AY2012-13 & AY2013-14

MSLQ self directed learning domains	Overall Mean (SD)	Pediatrics Mean (SD)	Medicine Mean (SD)
Intrinsic Value (pre ACE)	6.4 (.59)	6.4 (.62)	6.4 (.57)
Intrinsic Value (post ACE)	6.3 (.81)	6.3 (.95)	6.3 (.65)
Self-Efficacy (pre ACE)	5.1 (.86)	5.0 (.98)	5.2 (.73)
Self-Efficacy (post ACE)	5.4 (.85)**	5.3 (.98)	5.5 (.68)
Cognitive Strategy Use (pre ACE)	5.2 (.77)	5.3 (.79)	5.1 (.74)
Cognitive Strategy Use (post ACE)	5.4 (.86)	5.6 (.65)	5.1 (.99)
Self-Regulation (pre ACE)	4.9 (.83)	5.0 (.88)	4.8 (.79)
Self-Regulation (post ACE)	5.2 (.88)**	5.3 (.87)	5.1 (.89)

Note: **p <0.01

Overall, of the participating students (*n* = 48), the percentage of students who felt prepared for residency (combining 4 and 5 ratings) increased from 35 % (*n* = 17) to 67 % (*n* = 32) from before to after the ACE course (X^2^ = 5.5, df = 1, *p* < .02) (see Table 3 for details).

**Table 3:** ACE students’ completion of their individual learning plan goals and objectives and their perceptions of the ILPs

Survey items	Overall (*n*=48)	Completely achieved ILP goals and objectives	
		‘No’ (*n*=25)	‘Yes’ (*n*=23)
ILP helpful in structuring ACE learning objectives (Rate 1–5), no. (%)			
1-3 ratings	12 (25 %)	10 (40 %)	2 (9 %)
4-5 ratings	36 (75 %)	15 (60 %)	21 (91 %)
ILP helpful in organizing goals for career (Rate 1–5), no. (%)			
1-3 ratings	19 (40 %)	15 (60 %)	4 (17 %)
4-5 ratings	29 (60 %)	10 (40 %)	19 (83 %)

In terms of achievement of ILP goals and objectives, a total of twenty-three participating students (48 %) reported complete achievement of their ILP goals and objectives whereas twenty-five participating students (52 %) reported that they did not completely achieve their ILP goals and objectives at the end of the experience (see Table 4 for details).

**Table 4:** ACE students’ completion of their individual learning plan goals and objectives and their perceptions of preparedness for residency

Survey items	Overall (*n*=48)	Completely achieved ILP goals and objectives	
		‘No’ (*n*=25)	‘Yes’ (*n*=23)
Prepared for residency (Rate 1–5), no. (%) – Pre ACE			
1-3 ratings	31 (65 %)	17 (68 %)	14 (61 %)
4-5 ratings	17 (35 %)	8 (32 %)	9 (39 %)
Prepared for residency (Rate 1–5), no. (%) – Post ACE			
1-3 ratings	16 (33 %)	12 (48 %)	4 (17 %)
4-5 ratings	32 (67 %)	13 (52 %)	19 (83 %)

Of those students who reported that they completely achieved their ILP goals and objectives (*n* = 23), a higher percentage also reported feeling more prepared for residency (combining 4 and 5 ratings) after the ACE course (*n* = 19, 83 %). Of those who did not fully complete their ILP goals and objectives (*n* = 25), a significantly lower percentage (*n* = 13, 52 %) reported feeling more prepared for residency at the end of the ACE course (X^2^ = 5.1, df = 1, *p* < .03).

Additionally, a significantly higher percentage of students who had successfully achieved their ILP goals and objectives also indicated that the process of writing the ILP was helpful in structuring their ACE learning objectives (combining 4 and 5 ratings) (*n* = 21, 91 %) and organizing their learning goals (combining 4 and 5 ratings) in comparison to students who reported partial fulfillment of their ILP goals and objectives (*n* = 19, 83 %) (X^2^ = 6.3, df = 1, *p* < .01; X^2^ = 9.1, df = 1, *p* < .003 respectively).

Several themes emerged from the qualitative analysis of the ILPs and self-reflections. In relation to the ILPs, ‘Medical Knowledge’ was the core competency most frequently identified on the ILP as an area of concentration (90 %) whereas Professionalism was selected least frequently (4 %). From the self-reflections, students noted that they felt the process of writing the ILP allowed them to improve on certain knowledge and skills and pushed them to identify their own strengths and weaknesses. As far as career development was concerned, students felt that having a head start on goal development for internship improved their overall preparedness for residency training. In terms of challenges and limitations, students stated that it was difficult to determine specific and realistic goals appropriate to their level of training. Additionally, trying to implement self-assigned goals with the specific patient care opportunities they had within the limited time frame of the course proved challenging. In certain cases, students had difficulty achieving goals based on variable patient volume, clinical setting and resource restrictions. Samples of student written reflection statements are provided in Table 5.

**Table 5:** Examples of student written reflection statements

*Identifying Strengths and Weaknesses* • “Coming up with my own learning objectives was also good because it forced me to highlight some of my deficits and/or knowledge gaps, which is something I may not have done on my own.” *Preparation for Residency* • “It gave me a chance to balance the clinical duties and educational workload of a resident with same “real-life” responsibilities I will be facing as an intern. I feel more confident in terms of my communication as well as my ability to plan out and then accomplish my goals moving forward.” *Challenges and Limitations* • “The main challenge was realizing that I could not fully accomplish all my goals in a 1-month period. Developing my own history-taking style will take a lifetime, as will learning the intricacies of healthcare policies. Memorizing vaccinations will also require long-term practice. I overcame these challenges by telling myself to be patient and to just put in a decent effort each day without getting discouraged.” • “I think the ILP would have been much more powerful if students could have truly tailored their experience to their needs instead of assuming the tasks of a resident. For example, if a patient needs to be sent for a scan or a surgical procedure, the student may be able to stay with the patient and become a part of those procedures by observing or scrubbing in etc.”	

## Discussion

Results from our study indicate that inclusion of ILPs in undergraduate medical education improves SDL strategies among students. SDL is considered an integral component of each of the six ACGME core competencies. The ACGME has embraced the use of ILPs in graduate medical education programs to help learners assess their own educational needs and set goals to target those competencies [11, 12]. ILPs are useful tools to help fourth year medical students shape their learning experience as they prepare for their transition to residency training. Our results are consistent with the results reported by Shepard et al. [7] In both studies, students identified more learning needs in the competency of Medical Knowledge compared to Professionalism. This may be related to the short duration of both courses and the perceived student need to focus on their knowledge skills during that time period.

The SDL domains of self-efficacy and self-regulation both demonstrated significant improvements during the ACE course, indicating that our student’s beliefs in his/her ability to successfully achieve identified goals and tasks increased at the completion of the course. On the other hand, intrinsic value and cognitive strategy did not demonstrate significant improvement in our study. The intrinsic value of our students was considerably high at the beginning of the course and therefore we did not anticipate a further increase in this area. In terms of cognitive strategy, we believe that the short duration of the course might make it difficult to substantially change the way each individual student processes information. Any improvements in these SDL domains prior to residency are undoubtedly beneficial for development of successful future physicians.

In the qualitative analysis of self-reflections, students repeatedly identified faculty mentorship as an important component to success in achieving their learning goals. Each student received direct feedback from ACE course faculty at the beginning and mid-point of the course, but additional mentorship during the course varied and was not quantified by study investigators. The ACE course was required for all fourth year medical students, and offered within the disciplines of Internal Medicine, Pediatrics, Surgery, Obstetrics/Gynecology, Emergency Medicine, Neurology, Anesthesiology and Pathology. On several occasions, a student who was preparing for a residency program outside of those disciplines, ie. Radiology, might be assigned to Internal Medicine for their ACE course. In these instances, matching self-identified learning goals with the clinical offerings of the course was challenging, and may have negatively impacted successful achievement of learning goals. Comparing a concentrated week ILP to a distributed four week ILP, our ACE course directors felt that rather than giving students a consecutive week to work on their ILPs, it was more effective to spread the independent SDL days throughout the four-week rotation to allow for more flexibility in scheduling certain activities.

Lately, the educational benefit of the fourth year of medical school has been questioned with increasing numbers of medical schools considering the adoption of three-year curricula to meet the community workforce needs and reduce the economic burden on students. However, recent mixed-methods analyses of graduating fourth year medical students found that students uniformly assigned significant educational value to this final year of medical school [13–15]. The fourth year allowed them to gain clinical confidence and address fears about preparedness for residency training. In particular, senior students identified value in flexibility, individualization and exploration of practice in diverse settings after having been exposed to the core clerkships during their third year. The ILP tool provides a useful and workable framework for shaping this critical year of undergraduate medical training.

Furthermore, trainees would likely benefit from the use of the ILP throughout the entirety of their medical school training, as the process of building an ILP helps to hone self-reflective and SDL skills. There are several key transition points in undergraduate medical education where students should assess and evaluate their learning goals. Introductory ILPs can be developed when they enter professional education, and modified at critical junctures during their training, such as the transition from pre-clinical to basic clinical courses. Although in our study a majority of students identified the competency of Medical Knowledge as a learning goal, the ILP could be implemented as a vehicle to introduce students to the other core competencies of patient care, practice-based learning and improvement, interpersonal and communication skills and professionalism early in the course of their medical education. To maximize success, students need guidance to create and achieve their individual SMART learning goals from faculty advisors familiar with SDL and ILPs. For institutions considering the use of ILPs within undergraduate medical curricula, faculty development regarding SMART learning goals, ILP structure and SDL theory is essential.

## Conclusions

In this study, the use of the ILP was evaluated as it relates to a four-week clinical residency preparation course. Although our sample size is limited and the participants were self-selected, our study provides an early window into how ILPs can be utilized in brief curricular events with medical students. Students could use this framework to continually re-shape their learning goals and their career planning throughout the four years of medical school. Future research across other disciplines and institutions can help determine the broader applicability of the use of the ILPs in enhancing medical student self-directed learning and goal achievement.

## Abbreviations

AAMC: Association of American Medical Colleges
ACE: Advanced Clinical Experience
ACGME: Accreditation council on graduate medical education
ILP: Individualized learning plans
MSLQ: Motivated Strategies for Learning Questionnaire
SDL: Self-directed learning
SMART: Specific, measurable, achievable, realistic, timely
